# Complete genome sequence of *Paenibacillus* sp. PK1-4R (DSM 120655) isolated from the rhizosphere of *Brassica napus* in Rostock, Germany

**DOI:** 10.1128/mra.00124-26

**Published:** 2026-04-27

**Authors:** Philip Kirianki Ruciaka, Marie Chantal Lemfack, Boyke Bunk, Cathrin Spröer, Birgit Piechulla

**Affiliations:** 1 Institute of Biological Sciences, University of Rostock 9187, Rostock, Germany; 2 Leibniz Institute DSMZ, German Collection of Microorganisms and Cell Cultures 28351, Braunschweig, Germany; Indiana University, Bloomington, Bloomington, Indiana, USA

**Keywords:** *Paenibacillus*

## Abstract

We report on the complete genome sequence of the rhizobacterium *Paenibacillus* sp. PK1-4R. This might be a new species within the genus *Paenibacillus*, as evidenced by the taxonomic classification using the TYGS web server. It has a single chromosome (6,961,392 bp) but no plasmid.

## ANNOUNCEMENT

*Paenibacillus* sp. PK1-4R (DSM 120655) was isolated from the rhizosphere of *Brassica napus* in an agricultural field of Biestow (Rostock, Germany) (1). Biestow lies 4 km south to southwest of Rostock city (54°03′18.7″ N 12°05′30.1″ E). These agricultural fields have a long history of *Brassica napus* cultivation (2). Rhizosphere samples were dissolved in sterile double-distilled water, from which pure bacterial colonies were isolated on Luria–Bertani (LB) agar. A single *Paenibacillus* sp. PK1-4R colony was aerobically incubated overnight in 6 mL LB at 30°C.

The pellets were harvested by centrifugation (10,000 × *g*, 20 minutes). High-molecular-weight (HMW) genomic DNA was extracted using a commercial Monarch kit following the manufacturer’s instructions (NEB, Frankfurt, Germany). This allows gentle cell lysis, and the intact genomic DNA precipitates on glass beads. The quality of the HMW genomic DNA was checked on pulsed-field gel electrophoresis and was used to prepare libraries as described by Safaei and colleagues (3). The long-read PacBio SMRTbell (single-molecule real-time) library was prepared following the instructions of Pacific Biosciences (Menlo Park, CA, USA) in accordance with the procedure and checklist for preparing multiplexed microbial libraries using the SMRT Express Template Prep Kit 2.0 (4). Genomic DNA was sheared using G-tubes (Covaris, Woburn, MA, USA) according to the manufacturer’s instructions. Fragments smaller than 3 kb were removed in accordance with the procedure and checklist for preparing multiplexed microbial libraries using the SMRT Express Template Prep Kit 2.0 (4). PacBio sequencing was performed on the Sequel IIe system (Pacific Biosciences) taking one 15-hour movie.

The short-read library was prepared using the Illumina Tagment DNA enzyme and Small Buffer Kit, followed by sequencing using the MiSeq Reagent Kit v3 with 600 cycles (Illumina, USA). The quality control of raw read data were performed using SMRT Link (long reads) and FastQC v0.11.9 (short reads). The PacBio long reads were assembled using the microbial genome analysis protocol provided within SMRT Link v11.0.0 using a target genome size of 10 M, downsampled coverage of 100, and a maximum plasmid length of 300,000 bp as parameters (default). For error correction, non-trimmed Illumina short reads were aligned to the assembled PacBio long-read genome via BWA v0.7.17 (5) with subsequent variant calling using VarScan v2.4, generating single-nucleotide polymorphisms (SNPs) relative to the reference genome. For sequence correction, SNPs were incorporated into the reference genome, generating the consensus sequence (6). The contig was rotated to position 1 of the *dnaA* gene, annotated via NCBI PGAP (7), and analyzed via TYGS v389, a freely available in-house tool from DSMZ (https://tygs.dsmz.de), but also incorporating recently introduced methodological updates (8). The assembly statistics are summarized in Table 1.

**TABLE 1:** Genome features of *Paenibacillus* sp. PK1-4R

Features	Statistics
Number of Illumina reads	5,565,020
Read length (Illumina) (bp)	250
Number of PacBio reads	123,463
Mean read length (PacBio)	8,753
N50 read length (PacBio)	9,832
Mean coverage (chromosome, PacBio assembly)	155
GC content (%)	46.08
CDS	5,908
rRNAs (5S, 16S, 23S)	12, 12, 12
tRNAs	102
tmRNAs	1
BUSCO completeness score (%)	99.4
Contamination score (%)	0.13

## Data Availability

The genomic data of *Paenibacillus* sp. PK1-4R have been deposited at NCBI GenBank under project number PRJNA974575 and accession number CP126535.1. The raw reads can be accessed from the Sequence Read Archive (SRX30977238 [Illumina] and SRX30977239 [PacBio]). The SeqCode registry can be accessed from https://seqco.de/i:54906.
